# COGNITIVE FUNCTIONING AMONG GRANDPARENT CAREGIVERS: AN INTERGENERATIONAL PERSPECTIVE

**DOI:** 10.1093/geroni/igad104.2940

**Published:** 2023-12-21

**Authors:** Khushbu Patel, Katelyn Singer, Benjamin Katz

**Affiliations:** Virginia Tech, Blacksburg, Virginia, United States; Virginia Tech Graduate School, Blacksburg, Virginia, United States; Virginia Tech, Blacksburg, Virginia, United States

## Abstract

Grandparents have a unique bond with their grandchildren, given their position in the grandchild’s life. They may be a historian, mentor, playmate, caregiver, role model, advocate, and friend. These interactions may offer an opportunity for cognitive engagement, but the level of this engagement may in turn be linked to a grandparent’s cognitive status. For example, memory issues may challenge one’s ability to serve as a historian for a grandchild. To better understand the association of caregiving and engagement in activities with a grandchild on cognition, a secondary analysis was conducted to examine how cognitive functions differ by engagement time. Drawing from approximately 1,200 participants in the Health and Retirement Study who indicated activity engagement with grandchildren and who also completed the Harmonized Cognitive Assessment Protocol (HCAP) regression analyses were conducted examining the link between grandparent engagement with grandchildren and cognitive measures including Constructional Praxis Delayed, Backwards Counting, Raven’s Standard Progressive Matrices, Brave Man Delayed, and Digit Symbol Modalities. Results from the analyses revealed that Raven’s Matrices (P = 0.007), Backwards Counting (P = 0.041), and Constructional Praxis (P = 0.030), were most closely linked to activity engagement with grandchildren, providing preliminary evidence that working memory is closely linked to caregiving engagement. However, the results also revealed a bimodal distribution, suggesting that too little and too much caregiving were associated with worse cognition. These findings provide preliminary evidence that illustrates how interacting with and caregiving for grandchildren may be both a protective factor and a risk factor for older adults.

